# On the search for an intelligible comet assay descriptor

**DOI:** 10.3389/fgene.2014.00217

**Published:** 2014-07-17

**Authors:** Peter Møller, Steffen Loft, Clara Ersson, Gudrun Koppen, Maria Dusinska, Andrew Collins

**Affiliations:** ^1^Section of Environmental Health, Department of Public Health, University of CopenhagenCopenhagen, Denmark; ^2^Department of Biosciences and Nutrition, Karolinska InstitutetHuddinge, Sweden; ^3^Environmental Risk and Health Unit, Flemish Institute for Technological Research (VITO)Mol, Belgium; ^4^Environmental Chemistry (MILK), Health Effects Laboratory, Norwegian Institute for Air Research (NILU)Oslo, Norway; ^5^Department of Nutrition, Faculty of Medicine, University of OsloOslo, Norway

**Keywords:** biomonitoring, comet assay, DNA damage, ECVAG, FPG, tail moment

The comet assay has developed over the past 30 years and today, a variety of different DNA lesions and DNA repair can be measured by different versions of the assay (Collins, 2004). In the final step of the method, an image resembling a comet with a head (the nuclear core) and a tail (consisting of mainly single stranded DNA that has migrated out from the cell nuclei) is analyzed. The magnitude of the comet's DNA-tail provides information about the level of DNA lesions in the cell. The results from comet assay analyses are reported using different descriptors, the most frequently used being percentage of DNA in the tail (%T), tail length and tail moment (the product of %T and tail length). These descriptors can be reported in different ways, i.e., as means, medians or as distribution patterns. To compile the information on the migration of thousands of comets into a single value that is meaningful to convey to other researchers, is difficult. The solution has been practical and controlled by those researchers with the longest experience with the comet assay. In this opinion paper, we revisit the search for a commonly accepted descriptor for DNA damage measured by the comet assay. We define the “best” comet assay descriptor as a measurement that best describes the migration of DNA in each comet in the agarose, fits the distribution of comets in the gel, and conveys the technical measurement of comets as a descriptor that other researchers can understand. It should be emphasized that we do not embark on a mission to promote only one comet assay descriptor.

## What is the best descriptor of the DNA migration in the agarose gel?

**Figure 1:**
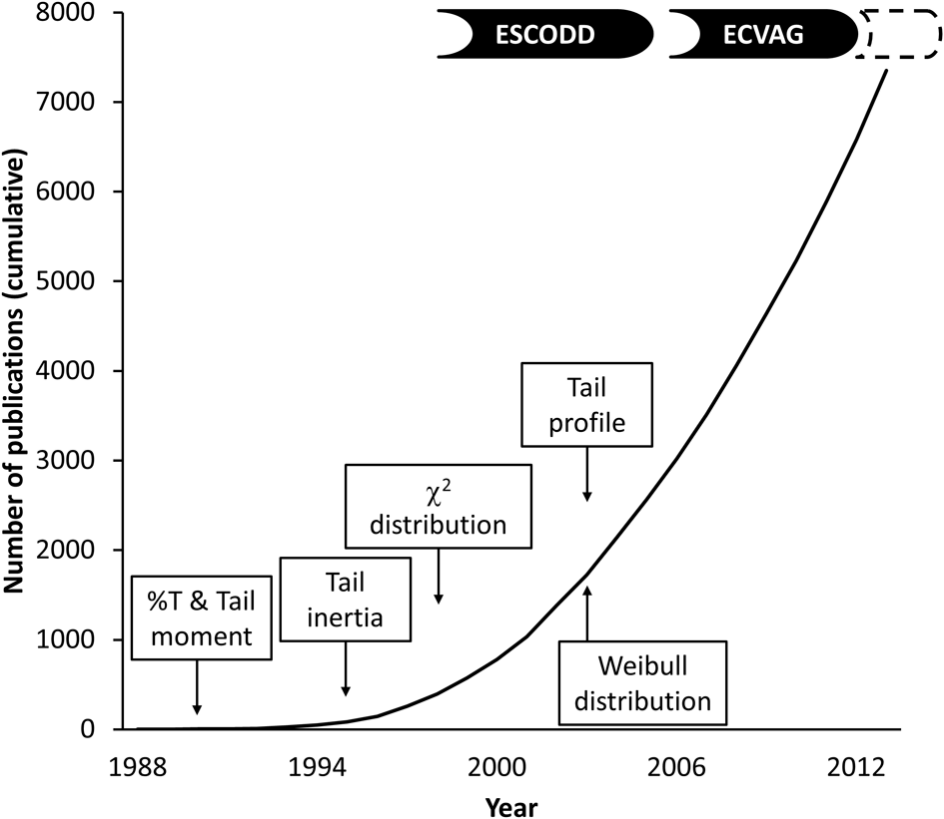
**Number of publications in PubMed using “comet assay” or “microgel electrophoresis” as search term and introduction of comet descriptors (see text for references)**.

Figure 1 outlines the number of comet assay publications and certain events in the development of comet quantification. The analysis of the comets (the final step of the assay) has progressed from the initial measurements of DNA migration (length) with an eyepiece micrometer, through semi-automatic image analysis of digitized comet images by software programs, to fully automatic systems with integrated tracking and image analysis of comets (Azqueta et al., 2013; Jackson et al., 2013). This equipment offers new possibilities to analyse comets in ways that were not previously possible. In addition, the fully (or semi-) automatic image analysis systems probably lift some of the restraints in the assay that are related to the manual measurement of each comet in the gels.

The majority of publications describing comet assay results adhere to the assumption that reliable information on the DNA migration in comets can be obtained by measuring %T. At an early stage, it was suggested that the tail moment gave a better description of the DNA migration than the more simple measurement of tail length or %T (Olive et al., 1990). An objection against tail moment has been that it is difficult to visualize the comets based on this descriptor. More refined ways of describing the DNA migration in the comets (e.g., “tail inertia” or “tail profile”) have not caught on Hellman et al. (1995); Bowden et al. (2003). This might have been due to the debate about tail moment or to the fact that these descriptors were not part of the software package for comet analysis at that time.

An alternative to the image analysis systems is the visual scoring system. This is based on a simple classification of the comets into (most commonly) five different classes, depending on the appearance of the comet (Gedik et al., 1992). This way to classify comets has been shown to be reproducible between laboratories scoring the same set of slides (Garcia et al., 2004). Although it is perceived as being less quantitative than computer-based image analysis systems, there are to the best of our knowledge no studies that have actually compared image analysis to visual scoring system in a systematic manner across laboratories.

## What is the best description of the distribution of comets in a sample?

Most laboratories measure the DNA migration by software systems in 50 or more randomly selected comets per gel in a minimum of 2 gels (Tice et al., 2000). This consensus is based on both practical and statistical considerations. For the visual classification system it has been common practice to score 100 comets per gel, which is probably because it is a relatively fast way of measuring the DNA migration and therefore one can afford the luxury of scoring more comets. However, it has been shown that increasing the number of scored comets per sample is associated with lower inter-sample variation and thereby with increased statistical power (Forchhammer et al., 2008; Sharma et al., 2012). These analyzed comets are not independent since they originate from the same sample (derived from a single experiment or measurement point). It is therefore common practice to regard the mean or median score of the comets originating from one sample as a single value. The damage (i.e., DNA migration) levels in the analyzed comets are mostly not normally distributed. Therefore, some researchers prefer to report the data as median rather than the mean. In our experience it makes little difference in the statistical analyses whether the underlying distribution of the comets has been described by the median or mean. In fact, it can be argued that both the median and mean are rather simple ways of describing the distribution. It has been shown that the underlying distribution of the comets can be described by a χ^2^-distribution (Bauer et al., 1998). The shape of the distribution is described as *number of degrees of freedom* and it is useful for the description of results that are subject to random variation. This is meaningful for the analysis of comet assay descriptors since there are heterogeneities within the gel, where comets with presumably the same level of DNA damage look different at certain positions of the gel. Nevertheless, this way of describing the underlying distribution of the comets has not been explored in detail, despite the fact that it provides a better fit of the data than the normal distribution. It has also been described that the underlying distribution can be fitted to a Weibull distribution, determined by two different descriptors, i.e., shape and scale (Ejchart and Sadlej-Sosnowska, 2003). This distribution has not been used in regular comet assay analyses, which is probably explained by the complexity of having to describe the level of DNA migration by two different values.

An often-raised question is whether the comet assay results can be analyzed by parametric tests when the underlying distribution is not normally distributed. Here it is important to keep in mind that statistical analysis is based on a descriptor for each sample (with its underlying distribution, e.g., the %T). The distribution of this descriptor score expressed as %T in e.g., peripheral blood mononuclear cells (PBMCs) from a group of humans, might be normally distributed or the data can be transformed to follow a normal distribution by for example log-transformation.

## What is the best comet assay result to report to other researchers?

There has been substantial debate over the years about which primary comet assay descriptor is the most relevant to use. Tail length has been discarded by many researchers since the maximal DNA migration is typically reached at low doses of exposure to DNA strand breaking agents (at least when analyzed with commonly used comet assay protocols). The debate about the use of %T or tail moment has diverted attention from the real issue of whether any of these descriptors are meaningful to researchers who are not familiar with the comet assay. These descriptors are quite seriously dependent on assay conditions (Azqueta et al., 2011; Ersson and Möller, 2011), and it would be more relevant to report DNA damage values after adjustment for the assay-specific conditions, typically by reference to standard curves. Nevertheless, reference values for DNA damage in terms of %T in PBMCs have been useful in human biomonitoring studies, which could be explained by the fact that most comet assay researchers in this specific field use similar assay conditions (Møller, 2006).

## What about the use of a reference standard?

As yet there is not a true standard in the comet assay like those that are used in chemical analyses. The use of reference standards has not yet been fully implemented, but it is recommended in published guidelines to use both positive and negative controls. There is no consensus about which agents should be used and an appropriate choice depends on the types of DNA-lesions that are measured. For instance, the detection of oxidatively damaged DNA requires a specific positive control for this endpoint. An advantage of ionizing radiation as positive control is that it can be applied both as positive control and calibration curve standard, since it is well-established how many DNA breaks a certain dose of ionizing radiation causes. The drawback is that it requires special equipment for the exposure.

The European Standards Committee on Oxidative DNA Damage (ESCODD) performed the first inter-laboratory trial to attempt a standardization of comet assay on human PBMCs. This project focused on oxidatively damaged DNA that can be measured by the comet assay as formamidopyrimidine DNA glycosylase (FPG)-sensitive sites. It was shown that the standardized results (lesions/10^6^ dG of FPG sensitive sites) were similar to results obtained with other techniques (i.e., the alkaline unwinding and alkaline elution assays) (ESCODD et al., 2005). The European Comet Assay Validation Group (ECVAG) subsequently looked further into approaches to reduce inter-laboratory variation in DNA damage by the use of calibration samples for standardization of comet assay descriptors (Møller et al., 2010). ECVAG settled on describing the DNA damage as lesions/10^6^ bp rather than lesions/10^6^ dG because the comet assay can be modified to measure various types of nucleobase lesions.

The first ECVAG trial assessed variation in the level of DNA strand breaks in coded cryopreserved calibration standards and test samples that had been distributed to 12 laboratories. This showed that all laboratories detected a dose-response relationship in coded samples, although there were differences in the reported values. The inter-laboratory coefficient of variation was 47% when the levels of DNA strand breaks were measured as %T or comet score, whereas it was 28% after transformation to lesions/10^6^ bp via the calibration curve (Forchhammer et al., 2010). The same analysis for FPG-sensitive sites showed that the participating laboratories could detect a dose-response relationship in coded cell samples. The conversion of %T to lesions/10^6^ bp increased the percentage of total variation explained by the inter-sample/subject variation from 49 to 73% (Johansson et al., 2010). A subsequent ECVAG trial looked into a standard comet assay protocol, but was only partly successful because some laboratories observed no difference in calibration curve samples and obtained negative values of FPG sensitive sites in human PBMCs (Forchhammer et al., 2012). ECVAG also showed that the overall variation of FPG-sensitive sites in the PBMCs could be partitioned into inter-laboratory (56.7%), residual (42.9%), intra-laboratory (0.2%) and inter-subject (0.3%) variation (Ersson et al., 2013). The most important finding in this trial was that the variation within each laboratory was relatively low.

Variation in DNA damage can be diminished by standardization of the primary comet assay descriptor using calibration samples. As highlighted by ComNet—a network of researchers using the comet assay in human biomonitoring studies—one of the challenges is to determine experimental factors that influence reliability and robustness of the comet assay as a biomonitoring tool (Collins et al., 2014). For, these kinds of studies, it is important to have low assay variability among laboratories. The number of scored comets could be an important determinant in this respect. But, maybe we also have to look more ahead and think of developing comet assay equipment with integrated calibration samples for standardization, and/or completely other scoring principles. Still some work to be done in the next 30 years!

### Conflict of interest statement

The Guest Associate Editor, Dr. Amaya Azqueta, declares that, despite having collaborated on a research project and publication with the authors, the review process was handled objectively. The authors declare that the research was conducted in the absence of any commercial or financial relationships that could be construed as a potential conflict of interest.
